# Endotoxin removal therapy with Polymyxin B immobilized fiber column: a single center experience from EUPHAS2 registry

**DOI:** 10.1038/s41598-023-44850-9

**Published:** 2023-10-16

**Authors:** Edoardo Forin, Giulia Lorenzoni, Ricard Ferrer, Massimo De Cal, Monica Zanella, Nicola Marchionna, Dario Gregori, Francesco Forfori, Anna Lorenzin, Vinicio Danzi, Claudio Ronco, Silvia De Rosa

**Affiliations:** 1 International Renal Research Institute of Vicenza Institute of Vicenza, San Bortolo Hospital, Vicenza, Italy; 2 Department of Anesthesiology and Intensive Care, San Bortolo Hospital, Vicenza, Italy; 3 Unit of Biostatistics, Epidemiology and Public Health, Department of Cardiac, Thoracic, Vascular Sciences and Public Health, University of Padova, Padova, Italy; 4 Shock, Organ Dysfunction, and Resuscitation Research Group (SODIR), Institut de Recerca Vall d’Hebron (VHIR), Barcelona, Spain; 5 Intensive Care Department, Hospital Universitari Vall d́’Hebron, Barcelona, Spain; 6 Department of Nephrology, Dialysis and Transplantation, San Bortolo Hospital, Vicenza, Italy; 7 Department of Surgical, Medical and Molecular Pathology and Critical Care Medicine, The University of Pisa, Pisa, Italy; 8 Centre for Medical Sciences - CISMed, University of Trento, Via S. Maria Maddalena 1, 38122 Trento, Italy

**Keywords:** Continuous renal replacement therapy, Diagnostic markers, Infection

## Abstract

Although the precise clinical indication for initiation of PMX-HA is widely debated in the literature, a proper patient selection and timing of treatment delivery might play a critical role in the clinical course of a specific subphenotype of septic shock (endotoxic shock). In light of this view, since 2019, we have introduced in our clinical practice a diagnostic-therapeutic flowchart to select patients that can benefit the most from the treatment proposed. In addition, we reported in this study our experience of PMX-HA in a cohort of critically ill patients admitted to our intensive care unit (ICU). We analyzed a single centre, retrospective, observational web-based database (extracted from the EUPHAS2 registry) of critically ill patients admitted to the ICU between January 2016 and May 2021 who were affected by endotoxic shock. Patients were divided according to the diagnostic-therapeutic flowchart in two groups: Pre-Flowchart (Pre-F) and Post-Flowchart (Post-F). From January 2016 to May 2021, 61 patients were treated with PMX-HA out of 531 patients diagnosed with septic shock and of these, fifty patients (82%) developed AKI during their ICU stay. The most common source of infection was secondary peritonitis (36%), followed by community-acquired pneumonia (29%). Fifty-five (90%) out of 61 patients received a second PMX-HA treatment, with a statistically significant difference between the two groups (78% of the Pre-F vs. 100% of the Post-F group, *p* = 0.005). In both groups, between T0 and T120, the Endotoxin Activity Assay (EAA) decreased, while the SOFA score, mean arterial pressure (MAP), and Vasoactive Inotropic Score (VIS) improved with no statistically significant difference. Furthermore, when performing a propensity score matching analysis to compare mortality between the two groups, statistically significant lower ICU and 90-day mortalities were observed in the Post-F group [*p* = 0.016]. Although in this experienced centre data registry, PMX-HA was associated with organ function recovery, hemodynamic improvement, and current EAA level reduction in critically ill patients with endotoxic shock. Following propensity score-matched analysis, ICU mortality and 90-day mortalities were lower in the diagnostic-therapeutic flowchart group when considering two temporal groups based on strict patient selection criteria and timing to achieve PMX. Further Randomised Control Trials focused on centre selection, adequate training and a flowchart of action when assessing extracorporeal blood purification use should be performed.

## Introduction

Sepsis is a severe condition characterized by a dysregulated and overwhelming inflammatory response resulting in circulatory, cellular and metabolic abnormalities and multiple organ dysfunctions. This complex multifactorial disease remains a significant health problem worldwide with very high mortality rates^1^: early identification and appropriate management are crucial. According to the last surviving sepsis campaign guidelines^2^, source control is the cornerstone of the treatment, while the microbiological cultures, the antibiotics administration and fluids/vasopressor resuscitation are essential elements associated with improved outcomes.

Based on the 28th Acute Disease and Quality Initiative (ADQI) suggested, in this setting, extracorporeal blood purification therapies have emerged as strategies and adjunct therapy able to perform immunomodulatory and/or kidney support. This allows removing the trigger (Pathogen Associated Molecular Patterns; PAMP) and/or providing immunomodulation by modifying the concentration of the damage-associated molecular patterns (DAMP). Moreover, if necessary, they can offer renal support the sequentially and possibly hybrid way in case of kidney failure^3^. Specifically, hemoperfusion involves the direct transit of the whole patient's blood over a sorbent bed or a reactor, usually contained in a cartridge, allowing immunomodulatory support^4^. Polymyxin B hemoadsorption (PMX-HA)^5^ cartridge, created by covalently immobilizing Polymyxin B, is a blood purification technique designed to bind and neutralize circulating endotoxin.

A clinically significant increase in circulating endotoxin levels might be observed during bactericidal antimicrobial therapy due to bacterial lysis, resulting in even further activation of inflammatory response^6^. Based on some recent evidence, patients with unresponsive septic shock may benefit from extracorporeal adsorption of circulating endotoxins as an adjunctive treatment^7,8^, especially those with a measured endotoxin activity assay^9^ (EAA) ranging from 0.6 to 0.9^10,11^.

Although some observational studies reported clinical benefits, particularly in specific subgroups of patients, more considerable randomized controlled trial results have been disappointing. Surviving Sepsis Campaign (SSC) guidelines 2016 did not recommend using blood purification techniques^1^. In the recent SSC guidelines 2021, the panel issued a weak recommendation against using PMX-HA^2^. A meta-analysis of all available PMX-HA RCTs reported a possible reduction in mortality (RR 0.87; 95% CI 0.77–0.98, low quality). However, sensitivity analyses challenged this result since after excluding trials with a high risk of bias, the risk ratio was 1.14 (95% CI 0.96–1.36). Moreover, after excluding trials published before 2010, PMX-HA was associated with a high mortality risk (RR 1.23; 95% CI 1.04–1.46). Overall, the quality of evidence is judged as low^2,12,13^.

As the precise clinical indication for initiation of PMX-HA is widely debated in the literature, we hypothesized that a proper patient selection and timing of treatment delivery might play a critical role in the clinical course of patients affected by septic shock^3^. In light of this view, since 2019, we have introduced in our clinical practice a diagnostic-therapeutic flowchart to select patients that can benefit the most from the treatment proposed. In addition, we reported our experience of PMX-HA in a cohort of critically ill patients admitted to our intensive care unit (ICU).

The aim of the present study is to report study our experience of PMX-HA in a cohort of critically ill patients admitted to our intensive care unit (ICU).

## Materials and methods

### Study design

The Early Use of Polymyxin B Hemoperfusion in Abdominal Septic Shock 2 (EUPHAS 2)^12,13^ project is a voluntary registry specifically conceived for evaluating and analyzing PMX-HA application in real clinical life. The EUPHAS2 observational, multicenter, multinational, prospective, web-based registry collects data concerning the use of PMX-HA in critically ill patients with septic shock, aiming to identify specific populations that may benefit from this therapy and provide proof of concept for future trials. We performed an observational, single-centre subgroup analysis of data from the EUPHAS2^14^ registry collected in our centre. In this subgroup, we included the 61 critically ill patients who were admitted to our ICU between January 2016 and May 2021 with a diagnosis of unresponsive endotoxic shock (defined as persistent hypotension (MAP < 65 mmHg) with organ dysfunction (Sequential Organ Failure Assessment (SOFA) > 7) despite fluid resuscitation, high-dose vasopressors) received PMX-HA treatment as per clinical indication of the attending physician.

In particular, although septic shock is characterized by at least a SOFA score greater than 11, data from EUPHAS Registry reported treated categories with SOFA at admission > 7. As confirmed by recent data from Japanese Diagnosis Procedure Combination (DPC) national inpatient database, PMX-HA treatment would significantly improve the survival of patients in the SOFA score categories of 7–9 and 10–12.

In 2019 we introduced in our clinical practice a flowchart for a rational approach and treatment of endotoxic shock patients based on evidence from the literature^3,10^. However, we evaluated two approaches to using PMX-HA and analyzed the factors related to mortality in propensity score-matched patients. Institutional Review Boards and the Ethical approved data collection and the study. Due to the retrospective nature of the study, informed consent was waived by Comitato etico della provincia di Vicenza (Sperimentazione 70/19).

### Patient management

All patients in this prospective observational registry received mechanical ventilation, extracorporeal blood purification therapies, antimicrobials, and any other treatment advised in patients with sepsis^1,2^. The definition of septic shock followed the consensus Sepsis-3 definition^1,2^. The Gram-negative aetiology of sepsis was suspected to be the source of infection or was proven by microbiological tests. Lipopolysaccharide (LPS) was assessed on peripheral blood samples by the neutrophil-dependent chemiluminescence-based EAA^14^. In all patients, PMX-HA was performed using a Toraymyxin® cartridge (Toray Medical Company, Tokyo, Japan) connected to the patient through a 10–12 French venovenous catheter inserted into the right or left femoral or internal jugular vein and the pump flow rate set at 80–120 mL/min. Each session of PMX-HA lasted 2 h. Unfractionated Heparin administration as an anticoagulant was set starting from a bolus of 3000 UI before the beginning of PMX-HA, followed by a continuous infusion during the treatment. The recommended dose of Heparin could be modified concerning the hemorrhagic risk of the patient^14^. The first 2-h treatment with PMX-HA was performed within 6 h from the ICU admission, followed by a second 2-h treatment after 24 h. A third treatment could be delivered if high endotoxin activity levels were still elevated and based on clinical decisions performed by the treating physician^14^.

### Measurements and parameters

Demographic variables were collected, including age, gender, Body Mass Index (BMI), past medical and surgical history, a clinical course in the department of origin and clinical severity at ICU admission, as reported in the EUPHAS Registry^12,13^. Suspected or proven sources of infection were recorded as well. The Simplified Acute Physiology Score (SAPS) II^15^ and Acute Physiology and Chronic Health Evaluation (APACHE) II^15^ scores assessed the clinical severity. Data regarding the PMX-HA treatment were recorded, such as the number of treatments and the anticoagulation modality^12,13^. Adverse events associated with PMX-HA were recorded and defined as. (1) worsening of sepsis/septic shock, in terms of tachycardia-heart rate (HR) > 100 bpm or HR increase > 10% HR pre-treatment *during 10*′* from the beginning of PMX-HA*, hypotension-MAP < 70 mmHg or PAM reduction > 10% MAP pre-*treatment during 10*′* from the beginning of PMX-HA*; (2) bleeding-every type of hemorrhagic complication, (3) cartridge clotting-coagulation of Polymyxin-B fiber cartridge. Clotting of the PMX cartridge was considered an adverse event as it caused a premature interruption of the treatment (limiting its benefit) and blood loss for the patient^12,13^. Concerning the 1-h bundle, we considered the type of antibiotic therapy and the amount of crystalloid and colloids infused^12,13^. Inflammatory biomarkers included procalcitonin and EAA (described in the following section). Clinical data were recorded before the PMX-HA commencement (T0) and 24 (T24), 48 (T48), 72 (T72), 96 (T96), 120 (T120) hours afterwards. The SOFA score assessed organ dysfunction^16^. Adequate perfusion was assessed by MAP, vasopressor requirement, and lactate levels. The dose of vasoactive/vasopressor agents was expressed as the Vasoactive Inotropic Score (VIS)^17^, a dimensionless variable calculated as (dopamine dose [μg kg^−1^ min^−1^] + dobutamine [μg kg^−1^ min^−1^] + 100 × epinephrine dose [μg kg^−1^ min^−1^] + 50 × levosimendan dose [μg kg^−1^ min^−1^] + 10 × milrinone dose [μg kg^−1^ min^−1^] + 10,000 × vasopressin [units kg^−1^ min^−1^] + 100 × norepinephrine dose [μg kg^−1^ min^−1^]) using the maximum dosing rates of vasoactive and inotropic medications (μg kg^−1^ min^−1^ or IU kg^−1^ min^−1^), where in all doses are expressed as μg kg^−1^ min^−1^. AKI was defined according to KDIGO criteria^18^. This study did not use the estimated glomerular filtration rate and urinary output criteria for AKI diagnosis and staging. Baseline serum creatinine (sCr) was calculated using the Modification of Diet in Renal Disease (MDRD) equation (back-estimation). In particular, in the absence of previous values, the baseline creatinine has been calculated as follow: GFR (Glomerular Filtration Rate) = (75/[186 × (age − 0.203) × (0.742 if female) × (1.21 if black)]) − 0.887). For each time point, sCr considered for AKI diagnosis was corrected for fluid balance according to recent evidence^19^. Simultaneously, we reported the need for sequential extracorporeal support and the modality of renal replacement therapy. Finally, we assessed the outcomes in terms of ICU and hospital length of stay, along with ICU, 28-day and 90-day mortalities.

### Endotoxin activity analysis

Blood endotoxin activity was measured by the EAA (Spectral Diagnostics Inc, Toronto, ON, Canada), which is a rapid (30 min) in vitro test that assesses neutrophil reaction to endotoxin via chemiluminescent response^9^.

EAA is a quick and easy diagnostic test based on a monoclonal antibody that identifies endotoxin. This method measures LPS activity based on the corresponding oxidative burst of primed neutrophils (complexes of an anti-endotoxin antibody and endotoxin) and is detected via the chemiluminescence method.

This technique allows measuring LPS in a short time (15–20 min): a rapid test for detecting endotoxemia in whole blood. EAA quantifies endotoxin levels through a relative scale ranking from 0 to 1. It is characterized by a sensitivity of 85.3%, a specificity of 44%, a negative predictive value of 98.6% for excluding Gram-negative infection and 94.8% for excluding all infections^20^. Marshall et al.^20^ demonstrated a progressive mortality increase related to endotoxin activity rising value. Based on post hoc exploratory analysis of the EUPHRATES trial, a strict patient selection criteria including high severity of illness and an endotoxin activity level as measured by EAA between 0.6 and 0.89, PMX use is associated with an absolute mortality benefit over sham patients of 10.7% at 28 days. For this reason, EAA greater than 0.6, considered the threshold to start the LPS removal.

### The diagnostic-clinical flowchart

**Figure 1:**
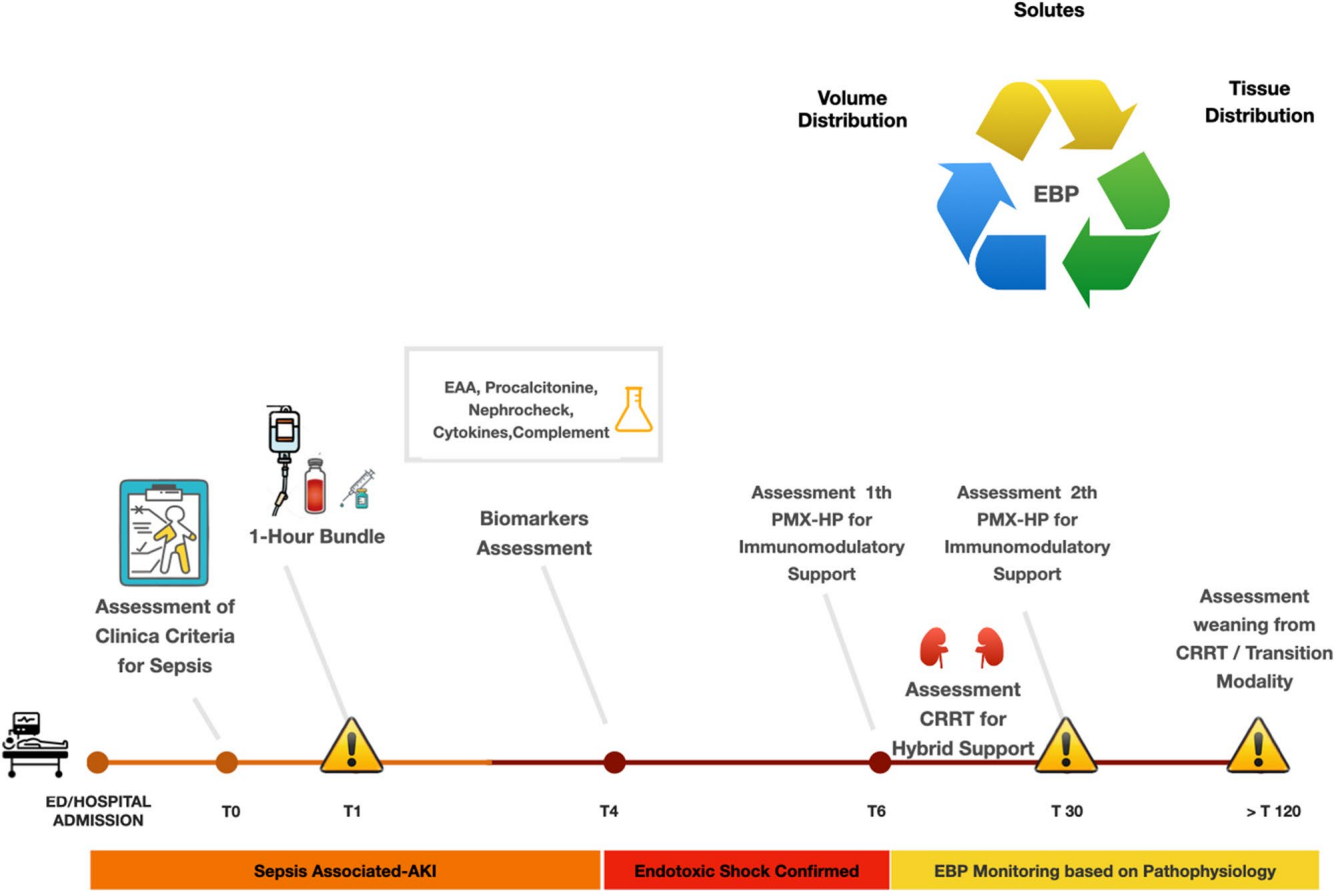
Diagnostic-clinical flowchart.

Since 2019, we have introduced a flowchart for a rational approach and treatment of endotoxic shock patients based on evidence from literature in our clinical practice. A diagnostic-clinical flowchart was presented in our current clinical practice, as shown in Fig. 1.

After the initial resuscitation for sepsis^1,2^, after performing the first-hour bundle, in the case of initiating vasopressor agents, EAA was tested within 4 h with a confirmation of endotoxic shock within a maximum of 6 h from the initiation of vasopressors.A Kidney Replacement Therapy (KRT) multidisciplinary team (just present before 2019), is activated whether specific precise criteria are fulfilled: serum lactate level > 2 mmol/L, vasoactive-inotropic score > 15, SOFA score > 7, procalcitonin levels > 0.05 ng/ml, 0.6 ≤ endotoxin activity < 0.9 or endotoxin activity < 0.6 and positive blood cultures.

The further and evident severe unresponsive shock (VIS > 35) and SOFA (> 15) and/or a high level of EA (equal or higher than 0.9), also based on data from Euphrates trial, was carefully evaluated and corroborated the extracorporeal endotoxin removal initiation. This protocol was also applied as a COVID-19-bedside strategy protocol for endotoxic shock.

At least 2-h treatments with PMX-HA at precise timing are then delivered, as carried out in most EUPHAS2 application protocols^12,13^, along with the kinetic trend of sequential EAA dosing (from T_0_ to T_120_ every 24 h)^21^. If a high endotoxin activity > 0.9 (associated with an unpredictable endotoxin concentration) is measured, the patient is excluded from the hemoperfusion treatment, as it could not modify the bad outcome, considering the clinical severity. If acute kidney injury (AKI) occurs (Kidney Diseases Improving Global Outcomes-KDIGO^18^ criteria ≥ 3), a sequential extracorporeal support for kidney and/or immunomodulatory support could be delivered, based on absolute indication to start CRRT. The KRT team, including intensivists, nephrologists, trained nurses and biologists, played a crucial role in performing, monitoring and assessing every step of the flowchart, as well as in the prevention and early management of any adverse event. In our centre, SOFA score is used to assess organ dysfunction in critically ill patients, including those with septic and endotoxic shock^21^.

### Statistical analysis

Descriptive statistics were reported as I quartile/median/III quartile for continuous variables and absolute numbers (percentages) for categorical variables. Wilcoxon-Kruskal–Wallis and Pearson Chi-squared tests were performed to compare continuous and categorical variables distribution, respectively. *p* values of repeated measures underwent the Benjamini–Hochberg procedure to account for the multiplicity of testing and control for false discovery rate. A propensity score weighting approach was employed to account for potential confounding related to the non-random allocation of the patients to the two interventions. Propensity scores were estimated using logistic regression, and the weights were trimmed at 90° quantile. Propensity scores were calculated considering lactates, SOFA score, APACHE II score and SAPS II Score. Covariate balance was evaluated using Standardized Mean Differences. A weighted logistic regression approach was adopted for binary outcomes. Results were reported as Odds Ratio, 95% Confidence Interval, and *p* value. Weighted Gamma models were employed to assess the effect of the intervention on continuous outcomes (hospital and ICU length of stay). The marginal effect was computed considering the partial derivatives of the marginal expectation. Results were reported as an average marginal effect, 95% Confidence Interval, and *p* value. Analyses were performed using R software^22^ within the packages rms, CBPS^23^ and WeightIt^24^ for propensity score weighting procedure estimation and margins^25^ for average marginal effect computation.

### Ethical statement

This study was approved by the Institutional Review Board (Comitato etico della provincia di Vicenza: Sperimentazione 70/19) according to Italian law. The study was conducted in accordance with the Declaration of Helsinki. Informed consent was waived due to the retrospective nature of the data.

## Results

The present retrospective analysis from the EUPHAS 2 registry, relating to a center expert in blood purification treatments, compared a population of patients treated with PMX-HA. In the statistical analysis, patients were divided into two groups based on the change in clinical practice. Before 2019, critically ill patients in septic shock were treated according to the 2016 SSC guidelines and treatment with PMX-HA was the prerogative of the single decision of the attending physician, in the absence of a multidisciplinary team, without considering timing, a dose and without the necessary presence of the EAA. The change in clinical practice included patient classification according to the 2019 guidelines, a well-defined protocol (Fig. 1) and the presence of a more experienced multidisciplinary team, with the inclusion of patients in septic shock with the positivity of EAA 0.6 ≤ endotoxin activity < 0.9 or endotoxin activity < 0.6 and positive blood cultures. The decision was made with a timing of 6 h after the first-hour bundle and the start of vasopressor drugs.

### Baseline patient characteristics

**Figure 2:**
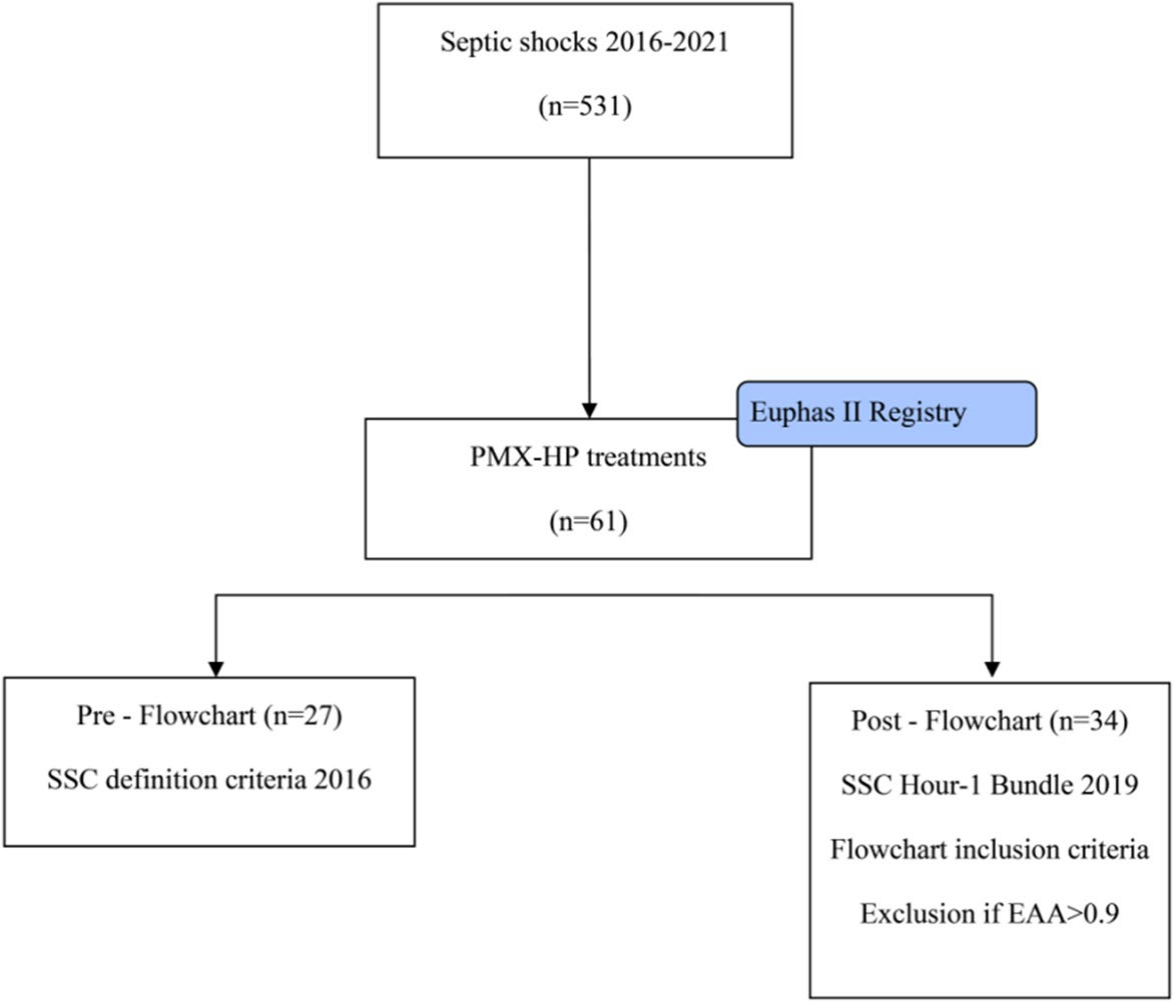
Study flowchart.

From January 2016 until May 2021, 61 patients were treated with PMX-HA out of 531 patients diagnosed with septic shock (Fig. 2) and were also followed for the development of AKI during ICU stay (Table 1). The Strengthening the Reporting of Observational Studies in Epidemiology reporting guideline checklist for observational studies was used for writing in this study. Patients were divided according to the diagnostic-therapeutic flowchart focused on identifying and timing endotoxic shock in two groups: Pre-Flowchart (Pre-F) and Post-Flowchart (Post-F). Demographic data of the 61 patients treated with PMX-HA are reported in Table 1. There were no statistically significant differences between the two groups. The median patient age was 70 [34, 89], and 42 (69%) were men. Remarkably, despite 31% of patients undergoing abdominal surgery before ICU admission and 36% having a diagnosis of secondary peritonitis, 29% presented community-acquired pneumonia. We identified gram-negative bacteria in most of the microbiological culture (N = 59.51%), followed by Gram-positive bacteria in (N = 31.27%), fungi (N = 11.9%), mixed (N = 37.70%) and no growth (N = 15.13%).

**Table 1:** Demographic data.

Characteristic	Entire Cohort N = 61	Pre-flowchart N = 27	Post-flowchart N = 34	*p* value^1^
Age (years), median (IQR)	70 (34,89)	72 (66, 78)	68 (61, 74)	0.11
Gender, n(%)				0.8
Male	42 (69)	19 (70)	23 (68)	
Female	19 (31)	8 (30)	11 (32)	
BMI (kg/m^2^), median (IQR)	24.3 (22.7, 27.8)	24.3 (22.7, 27.8)	25.8 (24.2, 29.2)	0.2
Type of suspected proven infection, n(%)				0.3
Wound infection	6 (10)	3 (11)	3 (8.8)	
Primary peritonitis:	8 (13)	2 (7.4)	6 (18)	
Secondary peritonitis	22 (36)	13 (48)	9 (26)	
Community-acquired pneumonia	18 (29)	5 (19)	13 (38)	
Surgical urinary tract infection	4 (7)	2 (7.4)	2 (5.9)	
VAP	3 (5)	2 (7.4)	1 (2.9)	
Main pathology, n(%)				0.2
None other than infection	27 (44)	9 (33)	18 (53)	
ALI/ARDS	6 (10)	3 (11)	3 (8.8)	
Atrial fibrillation	2 (3)	1 (3.7)	1 (2.9)	
Cholecystitis	4 (6)	4 (15)	0 (0)	
Colitis	2 (3)	1 (3.7)	1 (2.9)	
Colon cancer	1 (2)	0 (0)	1 (2.9)	
Intrabdominal abscess	2 (3)	1 (3.7)	1 (2.9)	
Intrabdominal perforation	9 (15)	5 (19)	4 (12)	
Intestinal occlusion	3 (5)	0 (0)	3 (8.8)	
Pancreatitis	3 (5)	2 (7.4)	1 (2.9)	
Trauma	1 (2)	1 (3.7)	0 (0)	
Thoracic aorta dissection	1(2)	0 (0)	1 (2.9)	
Comorbidities, n(%)				
Arrhythmia	6 (10)	4 (15)	2 (5.9)	0.4
Cancer	7 (11)	3 (11)	4 (12)	> 0.9
Diabetes	11 (18)	6 (22)	5 (15)	0.5
Hypertension	29 (49)	12 (44)	17 (50)	0.7
Hematologic malignancy	8 (13)	5 (19)	3 (8.8)	0.4
Obesity	4 (7)	1 (3.7)	3 (8.8)	0.6
Severe COPD	1 (2)	1 (3.7)	0 (0)	0.4
No comorbidities	44 (72)	21 (78)	23 (68)	0.4
Duration from hospital admission to septic shock (days)	1 (0, 36)	1 (0, 4)	2 (0, 10)	0.5
Durationfrom ICU admission to septic shock (days)	0 (0, 30)	0.0 (0.0, 1.0)	0.0 (0.0, 1.0)	0.5

^1^Wilcoxon rank sum test; Pearson's Chi-squared test; Fisher’s exact test.

ALI, acute lung injury; ARDS, acute respiratory distress syndrome; BMI, body mass index; COPD, chronic obstructive pulmonary disease; ICU, intensive care untit; PMX-HA, Polymyxin-B hemadsorption; VAP,ventilator-associated pneumonia.

### PMX-HA treatment characteristics

PMX-HA treatment characteristics are reported in Table 2. While the median days number from the ICU admission to the diagnosis of sepsis was 0 [IQR 0–30] (Table 1), the median days number from ICU admission to 1st PMX-HA treatment in the entire cohort was 1 [0, 30] (Table 2), with no statistically significant difference. Fifty-five (90%) out of 61 patients received a second PMX-HA treatment, with a statistically significant difference between the two groups (78% of the Pre-F vs. 100% of the Post-F group, *p* = 0.005). Considering the Pre-F group patients, the main reason for not performing the 2nd PMX-HA treatment was a clinical improvement (67%), followed by death (17%) and low life expectancy (17%). First, PMX-HA anticoagulation was significantly different in both groups: (a) the median heparin bolus was 3000 UI [3000, 3000] in the Pre-F group vs. 4000 UI [IQR 3000–5750] in the Post-F group (*p* 0.002); (b) the median Heparin Infusion dosage was 20 versus 16 UI/Kg/h (*p* < 0.001). Second PMX-HA treatment anticoagulation: (a) the median heparin bolus was not significantly different between the two groups, (b) 20 versus 15.5 UI/kg/h Heparin infusion respectively in the Pre-F and Post-F group (*p* = 0.003). In the overall population, 57 (93%) patients had no adverse events during 1st PMX-HA treatment compared to 53 (87%) during 2nd PMX-HA treatment. Within the two groups, during the 1st PMX-HA treatment, no adverse event was detected in 26 (96%) of Pre-F versus 31 (91%) of Post-F. In the Pre-F group, one patient had hypotension at connection compared to two in Post-F. Tachycardia was detected in one patient only in Post-F. During the 2nd PMX-HA treatment, no adverse event was detected in all Pre-F group patients, versus 32 (94%) of the Post-F group patients. Regarding 2nd PMX-HA treatment, tachycardia was registered in two patients of the Post-F group.

**Table 2:** Treatment characteristics.

Characteristic	Entire Cohort N = 61^1^	Pre-flowchart N = 27	Post-flowchart N = 34	*p* value^1^
Duration from septic shock to 1th PMX-HA (days)	1 (0, 36)	2.00 (1.00, 3.00)	1.00 (1.00, 2.00)	0.2
First PMX-HA, n(%)	61 (100)	27 (100)	34 (100)	–
Second PMX-HA, n(%)	55 (90.16)	21 (78)	34 (100)	0.005
Third PMX-HA, n(%)	3 (4.92)	0 (0)	3 (8.8)	0.2
Unfractionated Heparin bolus UI (1t^h^ PMX-HA)	3000 (3000, 6000)	3000 (3000, 3000)	4000 (3000, 5750)	0.002
Unfractionated Heparin infusion UI/kg/hr (1th PMX-HA)	20 (0, 20)	20 (20, 20)	16 (10, 20)	< 0.001
Adverse event (1th PMX-HA), n(%)				> 0.9
None	57 (93)	26 (96)	31 (91)	
Hypotension at connection	3 (5)	1 (3.7)	2 (5.9)	
Tachycardia	1 (2)	0 (0)	1 (2.9)	
Unfractionated Heparin bolus UI (2nd PMX-HA)	3000 (3000, 6000)	3000 (3000, 3000)	3000 (3000, 5750)	0.011
Unfractionated Heparin infusion UI/kg/h (2nd PMX-HA)	20 (0, 20)	20 (20, 20)	15.5 (10, 20)	0.003
Adverse event (2nd PMX-HA), n(%)				0.5
None	53 (87)	21 (100)	32 (94)	
Hypotension at connection	0 (0)	0 (0)	0 (0)	
Tachycardia	2(3)	0 (0)	2 (5.9)	
Reason for not performing the 2nd treatment, n(%)				> 0.9
Death	1 (17)	1 (17)	0 (0)	
Clinical improvement	4 (67)	4 (67)	0 (0)	
Low life expectancy	1 (17)	1 (17)	0 (0)	

^1^Wilcoxon rank sum test; Pearson’s Chi-squared test; Fisher’s exact test.

PMX-HA, Polymyxin-B hemadsorption.

### Endotoxic shock and PMX-HA treatment

Data regarding resuscitation, antibiotics and source control before PMX-HA are reported in Supplemental Table 1. Notably, the most two antibiotics used were Piperacillin/Tazobactam in 32 (52.5%) and Meropenem in 24 (39.3%) endotoxic shock patients. In addition, a new intervention for surgical source control was performed in 40 (65%) patients. PMX-HA and Endotoxic Shock Monitoring are shown in Table 3. The severity of patient's condition at admission, as evaluated in APACHE II, SAPS II score, and SOFA score, were 30 [IQR 10–50], 71 [IQR 24–108], 14 [IQR 8–21], respectively, with no significant statistical difference among the two groups. Firstly, in the overall population, median EAAprogressively decreased from 0.71 [IQR 0.5–0.93] at T0 to 0.46 [IQR 0.08–0.87] at T120, with no statistical difference between the two groups. Secondly, in the overall population, SOFA score progressively improved over the next 120 h following PMX-HA (median SOFA score was 14 [IQR 8–21] at T0 and 11 [IQR 2–22] at T120) with no statistically significant difference between the two groups.

**Table 3:** Polymyxin-B hemadsorptionand endotoxic Shock.

Characteristic	Entire Cohort N = 61	Pre-flowchart N = 27	Post-flowchart N = 34	*p* value^1^
APACHE II score, median (IQR)	30 (10, 50)	32 (28, 40)	28 (22, 34)	0.4
SAPS II score, median (IQR)	71 (24, 108)	64 (56, 82)	72 (53, 89)	0.9
EAA T_0_, units, median (IQR)	0.71 (0.5, 0.93)	0.70 (0.58, 0.79)	0.71 (0.64, 0.80)	0.7
EAA T_24_, units, median (IQR)	0.55 (0.04, 0.87)	0.39 (0.32, 0.46)	0.57 (0.48, 0.75)	0.4
EAA T_48_, units, median (IQR)	0.56 (0.18, 0.89)	NA (NA, NA)	0.56 (0.45, 0.66)	–
EAA T_72_, units, median (IQR)	0.55 (0.29, 0.9)	NA (NA, NA)	0.55 (0.41, 0.72)	–
EAA T_96_, units, median (IQR)	0.51 (0.2, 0.8)	NA (NA, NA)	0.51 (0.37, 0.60)	–
EAA T_120_, units, median (IQR)	0.46 (0.08, 0.87)	NA (NA, NA)	0.46 (0.36, 0.56)	–
PCT T_0_, ng/mL, median (IQR)	28.24 (0.3, 441.9)	41 (11, 115)	18 (5, 59)	0.4
PCT T_24_, ng/mL, median (IQR)	26.90 (0.3,429.5)	44 (13, 117)	25 (8, 47)	0.5
PCT T_48_, ng/mL, median (IQR)	18.61 (0.51, 367.2)	28 (16, 112)	12 (5, 27)	0.4
PCT T_72_, ng/mL, median (IQR)	10.57 (0.06, 206)	16 (11, 45)	7 (3, 13)	0.4
PCT T_96_, ng/mL, median (IQR)	5 (0.02, 180)	7 (2, 24)	4 (2, 8)	0.5
PCT T_120_, ng/mL, median (IQR)	2.5 (0, 55.05)	7 (1, 30)	2 (1, 4)	0.4
SOFA score T_0_, median (IQR)	14 (8,21)	14.0 (11.5, 15.5)	13.0 (12.0, 15.8)	0.8
SOFA score T_24_, median (IQR)	16 (3, 22)	16.0 (15.0, 19.0)	16.0 (10.2, 17.0)	0.4
SOFA score T_48_, median (IQR)	15 (2,21)	16.0 (13.0, 18.0)	14.0 (9.0, 17.0)	0.4
SOFA score T_72_, median (IQR)	13 (3, 21)	14.5 (10.2, 18.0)	13.0 (8.0, 16.0)	0.4
SOFA score T_96_, median (IQR)	12 (2, 22)	12.0 (11.0, 17.0)	11.0 (6.0, 13.0)	0.4
SOFA score T_120_, median (IQR)	11 (2, 22)	11.0 (7.0, 16.0)	10.0 (5.0, 13.0)	0.4
MAP T_0_, mmHg, median (IQR)	69 (48, 97)	70 (62, 76)	68 (65, 71)	0.8
MAP T_24_, mmHg, median (IQR)	71 (56, 96)	73 (70, 76)	70 (68, 75)	0.5
MAP T_48_,mmHg, median (IQR)	71.50 (54, 99)	72 (69, 87)	72 (70, 78)	> 0.9
MAP T_72_,mmHg, median (IQR)	72 (60,104)	75 (70, 82)	72 (70, 75)	0.5
MAP T_96_,mmHg, median (IQR)	72 (60, 104)	75 (70, 82)	72 (70, 74)	0.4
MAP T_120_,mmHg, median (IQR)	72 (60, 106)	71 (70, 87)	72 (70, 75)	> 0.9
VIS score T_0,_ median (IQR)	28 (14, 85)	25 (14, 35)	33 (20, 40)	0.4
VIS score T_24,_ median (IQR)	19 (0, 138)	21 (8, 33)	16 (10, 34)	0.8
VIS score T_48,_ median (IQR)	12 (0, 130)	13 (4, 19)	11 (0, 28)	> 0.9
VIS score T_72,_ median (IQR)	6 (0, 160)	8 (2, 18)	0 (0, 14)	0.5
VIS score T_96,_ median (IQR)	0 (0, 55)	6 (0, 10)	0 (0, 3)	0.4
VIS score T_120,_ median (IQR)	0 (0, 43)	0 (0, 10)	0 (0, 3)	0.4
Lactates T_0,_ mmol/L, median (IQR)	4 (0.5, 22)	4.0 (3.1, 6.6)	4.0 (3.0, 6.8)	0.8
Lactates T_24,_ mmol/L, median (IQR)	2.8 (0.8, 15)	3.00 (2.20, 5.00)	2.20 (1.25, 4.45)	0.4
Lactates T_48,_ mmol/L, median (IQR)	2.1 (0.4, 10)	2.45 (1.95, 3.80)	2.00 (1.25, 2.95)	0.4
Lactates T_72,_ mmol/L, median (IQR)	2 (0.7, 10.4)	2.00 (1.60, 2.82)	1.80 (1.30, 2.10)	0.4
Lactates T_96,_ mmol/L, median (IQR)	1.65 (0.6, 6)	2.00 (1.20, 2.70)	1.60 (1.10, 2.00)	0.5
Lactates T_120,_ mmol/L, median (IQR)	1.50 (0.2, 8)	1.50 (1.00, 2.00)	1.50 (1.00, 1.80)	0.7
Corticosteroids use, n (%)	43 (70.49)	22 (81)	21 (62)	0.4
Invasive MV, days, median (IQR)	11 (0, 77)	9 (4, 32)	12 (4, 20)	0.7
NIV, days, median (IQR)	0 (0, 6)	0.00 (0.00, 0.00)	0.00 (0.00, 0000)	0.8
Vasopressor requirement days median (IQR)	7 (0, 54)	8 (3, 16)	7 (4, 12)	0.8
28-days mortality, median (IQR)	15 (24.59)	8 (30)	7 (21)	0.7

^1^Benjamini & Hochberg correction for multiple testing.

APACHE II, Acute Physiologic Assessment and Chronic Health Evaluation (APACHE) II; EAA, endotoxin activity assay; MAP, mean arterial pressure; MV, mechanical ventilation; NIV, non invasive ventilation; PCT, procalcitonin; SAPS II, Simplified Acute Physiology Score II; SOFA, sequential organ failure assessment; VIS, vasoactive inotropic score.

In both groups, between T0 and T120, MAP and VIS improved with no statistically significant difference.

Lastly, the median lactate level decreased in both groups from T0 (4 [IQR 0.5–22] mmol/L) to T120 (1 [IQR 0–3] mmol/L) with no statistically significant difference between the two groups.

Inotropes and vasopressors in the overall population are shown in Supplemental Fig. 1.

### Acute kidney injury and sequential extracorporeal therapy

According to the KDIGO classification, among all 61 patients with endotoxic shock based on serum creatinine and urinary output variations, 50 (82%) experienced AKI without significant difference in both groups (Table 4). When considering only serum creatinine (70%) or urinary output variation (49%), the incidence of AKI decreases without significant differences in both groups. Forty-six patients (75.4%) after PMX-HA treatment started Sequential Extracorporeal Therapy by using CRRT for kidney and immunomodulatory support. Mainly, High-Volume Hemofiltration (HVHF) with high flux hemofilter was performed in 37 (80.4%) patients. Although without statistical significance in both groups, the use of HVHF was higher in the Pre-F group compared to the Post-F group, as shown in Table 4. Continuous Venovenous Haemodialysis (CVVHD) was performed in 8 (17.4%) patients, 6 with high flux membrane, 1 with high cut-off membrane and 1 with medium cut-off membrane. Mainly, 7 (33%) patients of the Post-F group were treated in CVVHD (6 with high flux membrane and 1 with medium cut-off membrane), while the single use of a high cut-off membrane in CVVHD was performed in the Pre-F group. Continuous venovenous hemodiafiltration (CVVHDF) with medium cut-off membrane was performed in only one patient in the Post-F group. Twenty-seven (64%) patients did not have a progression towards acute kidney disease (AKD): 13 (31%) were at stage 1, 1 (2.4%) at stage 2, and 1 (2.4%) at stage 3. This was more evident, although without statistical significance, in the Post-F group compared to the Pre-F group (69% vs. 54%, *p* value = 0.8). Renal Recovery was achieved in 20 (33%) patients, with a better percentage, although insignificant, in the Post-F group (61% vs. 55%).

**Table 4:** Acute Kidney Injury and Sequential Extracorporeal Support in Sepsis.

Characteristic	Entire Cohort N = 61	Pre- Flowchart N = 27	Post-Flowchart N = 34	*p* value^1^
AKI sCr KDIGO criteria, n(%)	43 (70)	20 (74)	23 (68)	> 0.9
AKI UO KDIGO criteria, n(%)	30 (49)	16 (59)	14 (56)	> 0.9
AKI UO e sCr criteria, n(%)	50 (82)	24 (89)	26 (76)	0.8
SETS (CRRT), n(%)	46 (75.4)	25 (93)	21 (62)	0.13
SETS after 1th PMX-HA, (n%)	38 (62.3)	23 (92)	15 (71)	0.5
SETS after 2nd PMX-HA, n (%)	36 (59)	21 (91)	15 (71)	0.5
MODALITY, n (%)				0.2
HVHF	37 (80.4)	23 (92)	14 (67)	
CVVHD	8 (17.4)	1 (4.0)	7 (33)	
CVVHDF	1 (2.17)	1 (4.0)	0 (0)	
Acute Kidney disease, n (%)				0.8
No	27 (64)	7 (54%)	20 (69)	
Stage 1	13 (31)	5 (38%)	8 (28)	
Stage 2	1 (2.4)	1 (7.7%)	0 (0)	
Stage 3	1 (2.4)	0 (0%)	1 (3.4)	
Renal recovery	20 (33)	6 (55%)	14 (61)	> 0.9

^1^ Benjamini & Hochberg correction for multiple testing.

AKD, acute kidney disease, AKI, acute kidney injury; CRRT, Continous Renal Replacement, Therapy; CVVHD, Continuous Venovenous Hemodialysis; CVVHDF, Continuous venovenous hemodiafiltration; KDIGO, Kidney Disease Improving Global Outcomes; HVHF, high volume high hemofiltration; PMX-HA, Polymyxin-B hemadsorption; sCr, serum creatinine; SETS, Sequential Extracorporeal Support in Sepsis, UO, urinary output.

### Short and long term outcomes

Regarding the outcomes (Table 3), the median ICU length of stay was 14 versus 16 days respectively, in Pre-F and Post-F groups, and the median hospital length of stay was of 34 and 28 days, respectively in Pre-F and Post-F groups), ICU mortality (52% vs. 29% respectively in Pre-F and Post-F group), 28-day mortality (30% vs. 21% respectively in Pre-F and Post-F group) and 90 days mortality (52% vs. 29% respectively in Pre-F and Post-F group). The propensity score weighting procedure resulted in a good balance of patients’ characteristics (Supplementary Fig. 2). The weighted analysis of the outcomes according to the propensity score weighting procedure showed a statistically significant lower ICU mortality (*p* = 0.0162) and 90 days mortality (*p* = 0.0162) in patients with the Post-F group (Table 5). Conversely, no differences were detected in the ICU length of stay (*p* value 0.1831) and the hospital length of stay (*p* value 0.8481).

**Table 5:** Results of the weighted regression models for the evaluation of the treatment effect on the outcomes of interest.

	Pre-flowchart	Post-flowchart	Estimate (95% CI)	*p* value
ICU length of stay	14 (6, 35)	16 (9, 24)	− 5.7056 (− 14.1051; 2.6940)	0.1831
Hospital length of stay	34 (15, 50)	28 (22, 49)	− 1.4852 (− 16.6801; 13.7097)	0.8481
90-day mortality	14 (52)	10 (29)	0.39 (0.18; 0.83)	0.0162
ICU mortality	14 (52)	10 (29)	0.39 (0.18; 0.83)	0.0162

The table reports, for continuous outcomes (hospital length of stay and ICU length of stay), the Average Marginal Effect (AME), together with the 95% Confidence Interval, and the *p* value. For the categorical outcomes (90-day mortality and ICU mortality), the table presents the Odds Ratio, together with the 95% Confidence Interval, and the *p* value. All the estimates referred to Flowchart versus No Flowchart use.

ICU, intensive care unit.

## Discussion

### Main findings

This study seeks to address an accurate clinical diagnostic approach to identify critically ill patients with a subphenotype of septic shock (endotoxic shock) with a timely assessment and management according to the Surviving Sepsis Campaign guidelines and following a diagnostic-clinical flowchart and with a support of multidisciplinary team to choose the right patient at the right time for a better patient outcome. In the overall population of refractory endotoxic shock due to abdominal and respiratory secondary infection, there was a decreasing in EAA, and an improvement of multiorgan dysfunction assessed by the SOFA, VIS and KDIGO scores at T120. No different EAA trend and SOFA score progression were observed among patients with abdominal and pulmonary infections. Following propensity score-matched analysis, ICU mortality and 90-day mortalities were lower in the Post-F group. In the Post-F group, the endotoxic shock was confirmed within 6 h from the norepinephrine requirement. PMX-HA commenced within 24 h of the diagnosis of endotoxic shock. A second cycle of this therapy was delivered 24 h afterwards.

### Diagnostic-therapeutic flowchart for refractory endotoxic shock

Endotoxic shock is an emergency but reversible condition if timely managed with appropriate and prompt administration of antimicrobial therapy and proper source control. Unfortunately, little is known about the clinical implications of antibiotic-induced endotoxin release that can cause adverse effects on patient outcomes^25^. Particularly, some classes of beta-lactam antibiotics, when used for the treatment of systemic Gram-negative infection, lead to markedly increased levels of free endotoxins, while treatment with carbapenems and aminoglycosides produces relatively low amounts of endotoxins^26^. xIn case of refractory endotoxic shock, a timely endotoxin removal^27^ and consequently immunomodulatory and anti-apoptotic effects^28^ could be performed using PMX-HA, which has been safely used to treat septic shock since 1994. Chang et al.^29^ and Terayama et al.^30^ demonstrated that PMX-HA treatment might reduce mortality in critically ill patients with severe sepsis or septic shock in specific disease severity subgroups. However, the use of PMX-HA is not standardized but rather highly variable between centres regarding EAA dosing, timing, number of PMX-HA treatments, monitoring and duration of treatments, but above all, patient selection^14,31–34^, as shown by data registry. This could have affected trial results, just compromised by design (i.e. underpower, unblinded, variable inclusion criteria, technical issues etc.). Since 2019, in our centre, a clinical diagnosis of sepsis and septic shock has followed a route based on a time-dependent five steps approach for identifying endotoxic shock supported by an efficient and well-trained multidisciplinary team, defined Renal Replacement Therapy (RRT) team. Precisely, the aforementioned five-step approach consists of the following steps: (1) identification of the patient in septic shock; (2) RRT Team alert; (3) Evaluation of the patient and execution of the EAA test; (4) Confirmation of endotoxic shock; (5) indication for PMX-HA treatment and management. This approach was also used in Coronavirus disease 2019 patients^21^. The RRT team assesses the patient over time, performing a dynamic prescription based on the patient's actual needs^21^. EAA test is routinely used to identify a specific patient population and monitor clinical conditions from baseline until 120 h from the start of treatment. Although there is no data in the literature, the endotoxin activity kinetics in our centre also helped the clinician monitor pharmacological and surgical source control. Further studies are needed to explore the relationship between endotoxin activity and the management of source control.

### Endotoxin activity, hemodynamic and organ function recovery

Between T0 and T120, the EAA, PCT and lactates were decreased in the overall population, consistent with the literature^14^. Further data regarding PMX-HA's beneficial effect on MAP and vasopressor dose are under investigation^35,36^. Fujimoriet al.^37^, found a significant association between PMX efficacy and a baseline SOFA score of 7–9 or 10–12. In our study, a progressive improvement in SOFA score was observed, also in an even higher baseline SOFA score, suggesting that the timely starting based on a careful assessment in most critically ill patients could improve the outcome.

### PMX-HA treatment

Although RCT studies have shown controversial results in patients with septic shock, mainly of abdominal origin, the appropriate number of sessions, the duration, and the timely initiation of PMX-HA administration are not documented and need to be defined. In our study, although the course of the treatment was 2 h in both groups, only in the Post-F group the number of sessions performed was always two. In addition, endotoxin kinetics and the regular assessment of endotoxic shock markers are critical tools for monitoring and tailoring the PMX-HA treatment. Considering also the high treatment costs and severity of the unresponsive endotoxic shock, based on VIS > 35 and SOFA > 15 and endotoxin activity > 0.9), extracorporeal endotoxin removal initiation should be carefully evaluated. Despite early clinical improvement that could lead to withholding the hemoperfusion treatment, we recommend performing a second treatment^3,21^. In addition, premature cartridge clotting is a significant concern during PMX-HA, leading to treatment discontinuation and waste of resources. In the Post-F group, a higher bolus of Unfractionated Heparin was administered, followed by a significantly lower infusion rate of Unfractionated Heparin, as the support given by the multidisciplinary team allowed a strict assessment and the early detection of clotting. Even though we do not have any data about it in the Euphas II registry, the infusion rate of Unfractionated Heparin was adjusted based on results of laboratory testing like activated partial thromboplastin time (aPTT) and activated clotting time (ACT), based on local anticoagulation protocol. In contrast to other previous studies^7,14,38^, no cartridge coagulation and treatment discontinuation due to early circuit coagulation were recorded, probably related to adequate assessment, according to local protocol, of both aPTT and ACT, performed by the multidisciplinary team, which allowed a more precise and early detection of those events, whose prevalence was similar to previous studies^7^. Other anticoagulation strategies are under investigation^39^.

### Acute kidney injury and sequential extracorporeal therapy

Sepsis-associated acute kidney injury (S-AKI) is a frequent complication of critically ill patients and is associated with unacceptable morbidity and mortality^40^. The incidence of sepsis or septic shock is high and increasing. However, Mehta et al.^41^ found that 40% of critically ill patients develop sepsis after AKI, suggesting that AKI may increase the risk of sepsis. In our study, S-AKI incidence was higher (82%), considering serum creatinine and urinary output variations using KIDGO criteria. This data could probably be related to the severity of endotoxic shock and the variability in the incidence based on the definition used^42^. Despite the in-hospital RRT requirement is strongly associated with hospital mortality, in our study, Sequential Extracorporeal Therapy in Sepsis (SETS) was performed. SETS is the application of different organ supports that may be combined sequentially to replace multiple organ dysfunctions. Before introducing the flowchart in our clinical practice, an assessment of sequential renal support therapy (RRT) was always performed before establishing PMX hemoperfusion treatment, so the flowchart formalized this. In particular, the absolute indications for renal replacement therapy (refractory hyperkalemia, refractory metabolic acidosis, fluid overload unresponsive to diuretics), which, if necessary, were both before and after PMX-HA treatment, were assessed.

In this case, extracorporeal support kidney and/or immune system (based on hemodiafilter or dialysis dose used) was applied, but the choice of kind of modality and hemodiafilter was based on clinical decision. In our center, HVHF was the modality most performed with a convective target dose (prescribed) greater than 35 ml kg^−1^ h^−1^^43–45^, but never over 50  ml kg^−1^ h^−1^. Clark et al.^44^ in a systematic review and Borthwick et al.^46^, in another systematic review and meta-analysis of randomized control trial (RCT), found insufficient evidence to suggest a therapeutic benefit for routine use of HVHF in sepsis other than on an experimental basis^46^.

Despite the absence of solid evidence in favour, in our center the multidisciplinary team prefer using this blood purification modality but with constant evaluation of prescription, with a maximum convective target dose of 50  ml kg^−1^ h^−1^ and rigorous adjustment of antibiotic therapy. In addition, in HVHF, transmembrane pressure drives vast deluges of plasma through the filter, allowing solute drag to pull more significant amounts of unpleasant substances from the patient. Furthermore, higher transmembrane pressures may increase the exposure of more membrane sites, improving ultrafiltration rates. Lastly, the membranes themselves may play a role in removing the cytokines by adsorption (i.e. direct deposition onto the membrane surface).

S-AKI has high potential to lead to AKD, or persistently reduced kidney function for at least seven but less than 90 days^47,48^. According to the findings^49^, patients who survived an AKI associated with sepsis often failed to return to baseline kidney function when they were discharged. Fortunately, in our study, many patients had not progressed towards AKD. The rest were classified most of all in AKD stage 1 (an increase of serum creatinine level to 1.5–1.9 times the baseline level) while the renal recovery was achieved in 33% of patients with a better percentage, not significant, in the Post-F group. The timely assessment and prompt performance of SETS probably allowed for the restoration of organ function and mitigated the damage. AKD stage may be a vital risk stratification tool for post-AKI care in patients surviving sepsis-associated AKI^49^. Further studies are needed to investigate the timing and the SETS in patients with endotoxic shock.

### Mortality

Despite SSC guidelines 2016 making no recommendation regarding the use of blood purification techniques, in the recent SSC guidelines 2021^2^, the panel issued weak guidance against the use of PMX-HA. Recently, as noted by^50,51^, although a meta-analysis of all available RCTs for PMX-HA reported showed a possible reduction in mortality, after excluding trials published before 2010, PMX-HA was found to be associated with a high mortality risk, and the quality of evidence was judged as low. However, our population’s weighted outcomes analysis according to the propensity score weighting procedure showed a statistically significant lower ICU mortality and 90-day mortality in patients with the Post-F group. An early and timely recognition of this subphenotype of septic shock supported by this flowchart and with adequate training of the personnel responsible for extracorporeal treatments could be the object of study of a future RCT to validate this defined approach and aimed at confirming these data on a larger population.

### Strengths and limitations

To the best of our knowledge, this is the first study that provides clinical data and specifically addresses the importance of using a clinical diagnostic flowchart for diagnosing and selecting patients for a specific blood purification treatment and sequential therapy. Moreover, based on our long experience in blood purification techniques, our data from daily clinical practice will allow external validation of our results. In addition, although the EUPHAS2^12,13^ registry still uses RIFLE criteria (Risk of renal dysfunction, Injury to kidney, Failure or Loss of kidney) for the standard classification for AKI, we decided to assess renal function based on the superiority in outcome prediction performance of KDIGO classification.

However, our article has some limitations. First, the descriptive nature of the study does not allow us to draw any conclusions on the use of the PMX-HA flowchart. Although we know that adequate mean arterial pressure is an essential prerequisite of tissue and organ perfusion during treating sepsis and that early titration of mean arterial pressure correlates with treatment success, other parameters may be helpful in combination MAP when performing perfusion assessment during extracorporeal blood purification.

Additionally, due to the Pre-Post design, some of the results observed could be a consequence of secular improvements in sepsis management and not only in implementing the flowchart and could be related also to a difference in the percentage of patients between both groups. However, it provides an exciting basis for directing future studies of randomized control trials with an adequate sample size and which provides for a proper selection and training of the centres involved. In addition, we did not report data-specific timing expressed in hours and on SETS characteristics (i.e. data on specific absolute indications for renal replacement treatment) because they were not present as a variable in the EUPHAS 2 registry.

In addition, we did not report differences between other treatments (e.g. antibiotics, vasopressors). We did not better investigate patients’ heterogenicity regarding the site of infections, exploring if PMX-HA hemadsorption work better in respiratory tract infections than abdominal infections.

## Conclusion

Although in this experienced centre data registry, PMX-HA was associated with organ function recovery, hemodynamic improvement, and current EA level reduction in critically ill patients with endotoxic shock. Following propensity score-matched analysis, ICU mortality and 90-day mortalities were lower in the diagnostic-therapeutic flowchart group when considering two temporal groups based on strict patient selection criteria and timing to achieve PMX, according to recent consensus. Further Randomised Control Trials focused on centre selection, adequate training and a flowchart of action when assessing extracorporeal blood purification use should be performed.

### Supplementary Information

Supplementary Information.

## Data Availability

The data supporting this study’s findings are available from the corresponding author upon reasonable request.

## Abbreviations

AKD: Acute kidney disease
AKI: Acute kidney injury
APACHE II: Acute physiology and chronic health evaluation II
EAA: Endotoxin activity assay
CVVHD: Continuous venovenous haemodialysis
CVVHDF: Continuous venovenous hemodiafiltration
EUPHAS 2: The early use of Polymyxin B hemoperfusion in abdominal septic shock 2
DAMP: Damage associated molecular patterns
GFR: Glomerular filtration rate
HVHF: High-volume hemofiltration
HR: Heart rate
ICU: Intensive care unit
KDIGO: Kidney diseases improving global outcomes
KRT: Kidney replacement therapy
LPS: Lypopolysaccaride
MAP: Mean arterial pressure
MDRD: The modification of diet in renal disease
PAMP: Pathogen associated molecular patterns
PMX-HA: Polymyxin B hemoadsorption
RRT: Renal replacement therapy
Pre-F: Pre-flowchart
Post-F: Post-flowchart
RIFLE: Risk of renal dysfunction, injury to kidney, failure or loss of kidney
RCT: Randomized controlled trial
SAPS II: Simplified acute physiology score
SETS: Sequential extracorporeal therapy in sepsis
SOFA: Sequential organ failure assessment
sCr: Serum creatinine
VIS: Vasoactive inotropic score
