# Supplementary material for: Endotoxin removal therapy with Polymyxin B immobilized fiber column: a single center experience from EUPHAS2 registry
Source: Sci Rep. 2023 Oct 16;13:17600. doi: 10.1038/s41598-023-44850-9 (PMC10579294; doi:10.1038/s41598-023-44850-9)
Supplement: Supplementary file 1 — Supplementary Information. [file 41598_2023_44850_MOESM1_ESM.docx]

Supplemental Material

Supplemental Figure 1. Inotropes and vasopressors in overall population


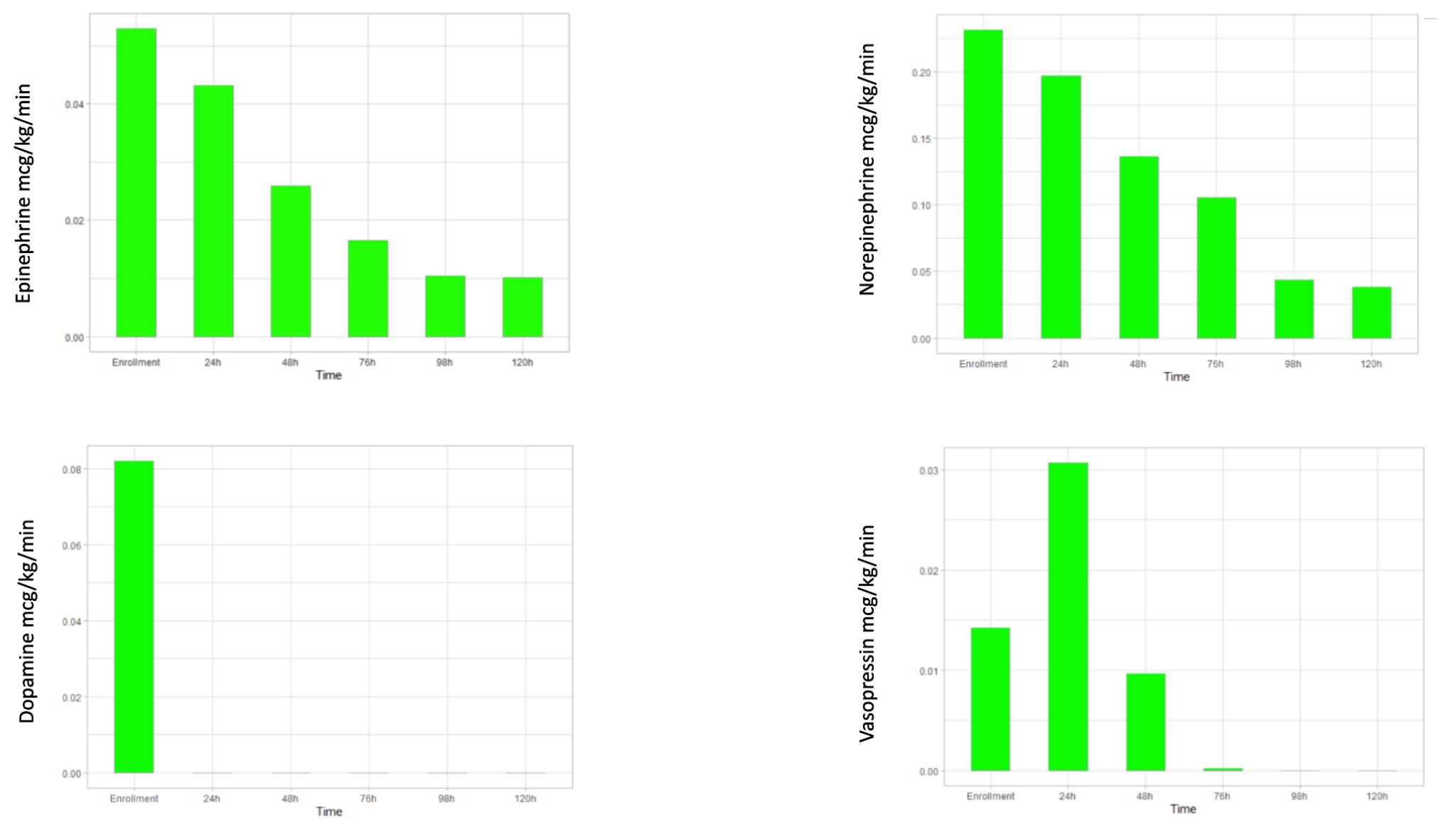


Supplemental Figure 2. Propensity score - balance of covariates

*
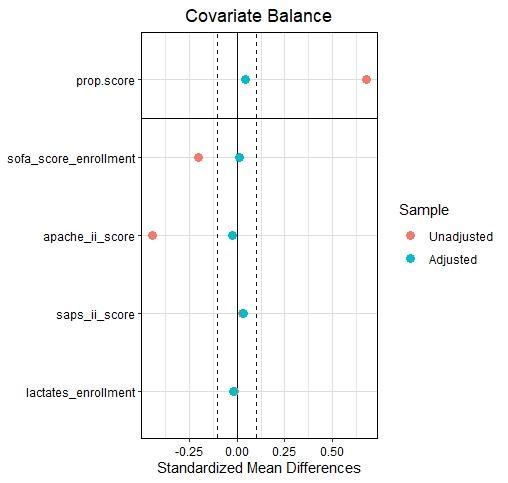
*

Supplemental Table 1. Resuscitation, Antibiotic and Source Control before PMX-HA

| Characteristic | Entire Cohort  N=61 | Pre- Flowchart  N = 27 | Post-Flowchart  N = 34 | p-value^1^ |
| --- | --- | --- | --- | --- |
| Lactate, _,_mmol/L, median (IQR) | 4.4 (0.6,18) | 4.5 (3.2, 6.4) | 4.2 (2.5, 7.0) | 0.7 |
| Blood culture, n (%) | 60 (98.4) | 26 (96) | 34 (100) | 0.4 |
| Linezolid, n (%) | 14 (22.9) | 6 (22) | 8 (24) | >0.9 |
| Piperacillin tazobactam, n (%) | 32 ( 52.5) | 16 (59) | 16 (47) | 0.3 |
| Ciprofloxacin, n (%) | 2 (3.3) | 1 (3.7) | 1 (2.9) | >0.9 |
| Gentamicin, n (%) | 1 (1.6) | 1 (3.7) | 0 (0) | 0.4 |
| Meropenem, n (%) | 24 (39.3) | 10 (37) | 14 (41) | 0.7 |
| Imipenem, n (%) | 1 (1.6) | 1 (3.8) | 0 (0) | 0.4 |
| Metronidazole, n (%) | 3 (4.9) | 1 (3.8) | 2 (5.9) | >0.9 |
| Colistin, n (%) | 1 (1.6) | 1 (3.7) | 0 (0) | 0.4 |
| Tigecycline, n (%) | 13 (21.3) | 7 (26) | 6 (18) | 0.4 |
| Ceftazidime, n (%) | 2 (3.3) | 1 (3.7) | 1 (2.9) | >0.9 |
| Levofloxacin, n (%) | 3 (4.9) | 2 (7.4) | 1 (2.9) | 0.6 |
| Daptomycin, n (%) | 1 (1.6) | 1 (3.7) | 0 (0) | 0.4 |
| Teicoplanin, n (%) | 1 (1.6) | 0 (0) | 1 (2.9) | >0.9 |
| Vancomycin, n (%) | 1 (1.6) | 1 (3.7) | 0 (0) | 0.4 |
| Fluconazole, n (%) | 3 (4.9) | 0 (0) | 3 (8.8) | 0.2 |
| Total amount of crystalloids infused, L, median (IQR) | 1.5 (0.5,4.1) | 1.00 (1.00, 1.50) | 1.50 (1.00, 2.00) | 0.3 |
| Total amount of albumin infused,mg/kg, median (IQR) | 20 (2,40) | 20 (16, 20) | 5 (4, 22) | >0.9 |
| Surgical Source Control (New Intervention), n (%) | 40 (65) | 5 (19) | 7 (21) | 0.8 |
| 1 Benjamini & Hochberg correction for multiple testing | | | | |
